# A Review of Temperature Effects on Membrane Filtration

**DOI:** 10.3390/membranes14010005

**Published:** 2023-12-24

**Authors:** Bochao Xu, Wa Gao, Baoqiang Liao, Hao Bai, Yuhang Qiao, Walter Turek

**Affiliations:** 1 Department of Civil Engineering, Lakehead University, 955 Oliver Road, Thunder Bay, ON P7B 5E1, Canada; bxu6@lakeheadu.ca; 2 Department of Chemical Engineering, Lakehead University, 955 Oliver Road, Thunder Bay, ON P7B 5E1, Canada; 3 Department of Mechanical Engineering, Lakehead University, 955 Oliver Road, Thunder Bay, ON P7B 5E1, Canada; hbai@lakeheadu.ca (H.B.); yqiao5@lakeheadu.ca (Y.Q.); 4 Environment Division, City of Thunder Bay, Victoriaville Civic Centre, 111 Syndicate Ave S., Thunder Bay, ON P7E 6S4, Canada; walter.turek@thunderbay.ca

**Keywords:** membrane filtration, membrane cleaning, membrane fouling, membrane structure, water temperature

## Abstract

Membrane technology plays a vital role in drinking water and wastewater treatments. Among a number of factors affecting membrane performance, temperature is one of the dominant factors determining membrane performance. In this review, the impact of temperature on membrane structure, fouling, chemical cleaning, and membrane performance is reviewed and discussed with a particular focus on cold temperature effects. The findings from the literature suggest that cold temperatures have detrimental impacts on membrane structure, fouling, and chemical cleaning, and thus could negatively affect membrane filtration operations and performance, while warm and hot temperatures might expand membrane pores, increase membrane flux, improve membrane chemical cleaning efficiency, and interfere with biological processes in membrane bioreactors. The research gaps, challenges, and directions of temperature effects are identified and discussed indepth. Future studies focusing on the impact of temperature on membrane processes used in water and wastewater treatment and the development of methods that could reduce the adverse effect of temperature on membrane operations are needed.

## 1. Introduction

Membranes have been universally utilized in water and wastewater treatments in recent decades. The extensive application of membrane technology has demonstrated the merits of high contaminant removal efficiency, small footprint, simple operation, and less chemical usage [1]. Therefore, membrane research has been constantly focused on the innovation and optimization of membrane technology.

While the advantages of membrane technology are predominant, many factors, such as feedwater characteristics, pH, temperature, flow rate, membrane materials, and configuration, could influence the performance of the membrane [2,3,4,5,6,7]. In most cases, membrane permeability/flux loss during operation becomes inevitable, potentially owing to membrane fouling and structural changes [6,8]. Although numerous studies have investigated causes and solutions for the fouling problems in membrane filtration [2,3,4,5,9,10,11], the influences of temperature on membrane fouling and structural changes have not been well studied.

**Figure 1:**
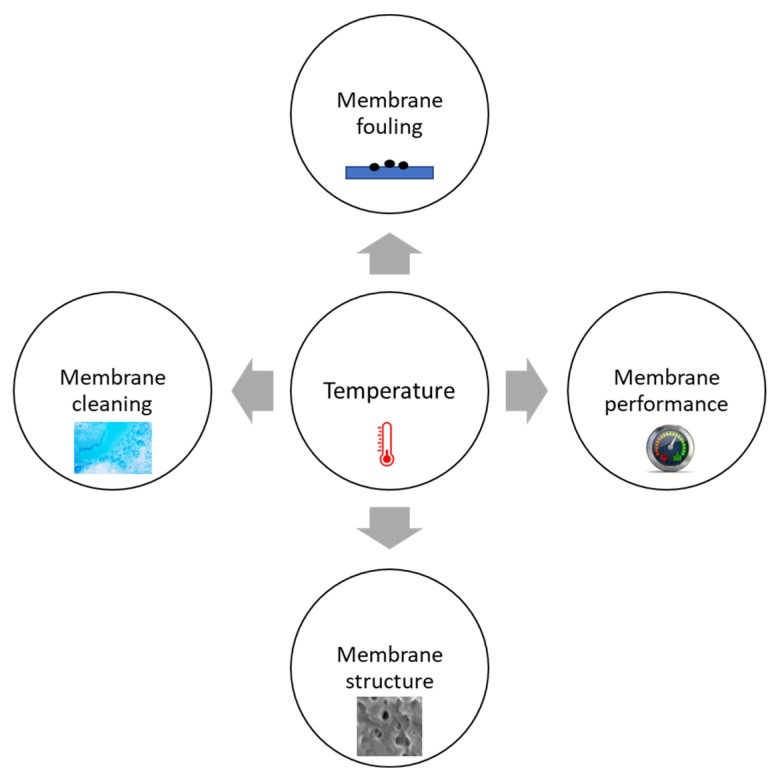
Impacts of temperature on membrane systems.

Temperature is an intrinsic property of feed water and thus plays a significant role in determining the efficiency and performance of membrane filtration plants. For example, temperature affects water viscosity and thus could reduce membrane permeability and/or flux at cold temperatures [12,13], which has been a significant challenge for the maintenance and operation of drinking water and wastewater treatment plants in cold regions [14,15,16]. Furthermore, the performance of biological wastewater treatment in membrane bioreactors (MBRs) is closely related to the operational temperature [17,18]; membrane structure and fouling rates are affected by the operating temperature of the membrane systems, such as various MBRs used in wastewater treatment [19,20]. In addition, the water temperature significantly impacts treatment performance, especially in cold regions with extreme seasonal temperature variations [6,14,15,16,19]. Figure 1 illustrates the impacts of temperature on membrane systems.

This review focuses on the impact of temperature (both cold and hot temperatures) on membrane fouling, flux and/or permeability, chemical cleaning, and membrane structure and integrity of membrane systems used in drinking water and wastewater treatment. 

## 2. Temperature Impacts on Membrane Fouling

Membrane fouling is a crucial hindrance to water treatment using membrane filtration. Therefore, numerous studies have investigated the effects of temperature on membrane fouling (Table 1). These research results indicated that temperature could either facilitate or mitigate the problems of fouling due to the foulant property shifting incurred by temperature change and fluctuation.

For instance, decreasing the operating temperature of MBRs to lower than 10 °C dramatically increased extracellular polymeric substances (EPS), soluble microbial products (SMP), proteins, polysaccharides and soluble chemical oxygen demand (COD) concentrations in the supernatants of MBR systems, and these biopolymers caused membrane fouling [23,25]. As a result, the capillary suction time (CST) and diluted sludge volume index (DSVI) were also higher at lower temperatures, making the sludge hard to settle and dewater. Thus, higher membrane fouling rates were reported at low temperatures (8.7–10 °C) than at high temperatures (19.7–20 °C) [24,25].

Gao et al. [18] investigated how temperature shock impacted the performance and microbial community structure on a submerged anaerobic membrane bioreactor (SAnMBR). Their research results indicated that microbial species and richness in the sludge were diverse in the different operating temperatures, with the high temperature stimulating the sludge layer accumulation but decreasing the layer thickness and total biomass aggregation. Furthermore, they found that high/warm-temperature shock benefited SAnMBR and promoted the biogas-producing rate, but increasing the water temperature from 37 °C to 45 °C resulted in the breaking of the sludge flocs into smaller pieces, which then formed the cake layer in the bulk sludge. In addition, the colloidal particles, SMP, and bound EPS accumulated, and there was additional fouling resistance with high-temperature shock [18].

On the contrary, although the biomass had lower growth rates at the lower temperature (10 °C), the sludge layer was thicker, thus detrimental to the permeation flux and salt retention ability [27]. A pair of abnormal temperatures, 40 °C and 60 °C, were tested to distinguish biofouling developments in a membrane distillation system researched by [29]. They found that high temperature (60 °C) caused significant flux decline and deposited protein-like matter and salt crystals on the membrane surface. Notably, the structures of microbial communities within the foulants at 60 °C dramatically differed from those at 40 °C.

Rong et al. [20] reported that when an anaerobic membrane bioreactor (AnMBR) was implemented to purify municipal wastewater, the membrane fouling condition deteriorated at a temperature of 15 °C, resulting from the accumulation of microbial products and inorganic foulants accompanied by increasing water viscosity. Hube et al. [32] investigated the temperature impact on a gravity-driven microfiltration membrane reactor treating domestic wastewater and revealed that more foulants with larger sizes within the cake layer accumulated on the surface of the membrane at 22 °C compared to that at 8 °C. They concluded that the membrane had similar cake resistance under the two conditions (8, 22 °C) since no cleaning was presented, while the membrane at room temperature (22 °C) had remarkably higher cake resistance than at the low temperature of 8 °C when there was periodic cleaning with the water temperature at 50 °C. However, the temperature variance did not influence foulant contents and permeate quality in this reactor [32]. In addition, Tao et al. [33] reported that polysaccharides positively correlated with hydraulically reversible resistance, while polysaccharides and low-molecular-weight substances induced hydraulically irreversible resistance in hollow fibre ultrafiltration MBRs used to treat municipal wastewater. They also showed that the total membrane resistance increases caused by fouling and intrinsic resistance were 55% and 122% at 14 °C and 8 °C, respectively, compared to the resistance at 20 °C [33].

A forward osmosis (FO) membrane system was used to purify source water with an elevated temperature of 50 °C [26]. The elevated temperature decreased internal concentration polarization and viscosity, which resulted in increased permeable flux. The FO membrane also encountered less fouling due to the presence of more soluble organic substances and the boosted back diffusion of organics on the membrane surface [26]. In Alresheedi and Basu’s [11] study of a ceramic ultrafiltration system, the lowest filtration fluxes were observed at 5 °C, and the highest fluxes were at 35 °C compared to the fluxes at 20 °C, owing to the shifts in water viscosity and membrane material resistances. The natural organic matter (NOM) retention efficiencies were 10% higher at 5 °C and 16% lower at 35 °C compared to the efficiency of 78% at 20 °C because the lower temperature changed the fouling layer structures and enlarged NOM size. Besides higher flux decreasing rates and NOM retention efficiencies, lower temperature also led to lower hydraulic and chemical cleaning efficiencies [11]. Both high viscosity and exacerbated fouling conditions contributed to half the increase in transmembrane pressure (TMP) at the low temperature (5 °C) for ceramic ultrafiltration. In addition, the low temperature could induce more irreversible foulants, which blocked the inside of membrane pores, than room temperature and 35 °C [13].

In a distillation study with a graphene oxide (GO) coated calcium sulphate reverse osmosis (RO) membrane, Ashfaq et al. [30] found that the high temperatures in warm regions like Arabian countries resulted in severe membrane scaling and high resistance by boosting the precipitation reaction. They also revealed that the morphology of the scaling was distinctive under different temperatures, and the membrane tended to be more hydrophilic after scaling [30]. Among scaling foulants, calcium sulphate was more likely to form a sizeable needle-shaped crystal at high temperatures up to 80 °C and severely foul the membrane [31].

To summarize, the published research (Table 1) indicated that feedwater/operating temperature affected membrane fouling. The impact of low temperatures and high temperatures, however, were different. Low feedwater/operating temperatures in the range of 5 to 15 °C and operating temperature reductions within a specific temperature range (5 to 35 °C) increased membrane fouling. MBRs operating at lower temperatures caused stronger deflocculation, which led to smaller sludge particles and EPS generation. At the same time, slower biodegradation of COD resulted in more organic matter, which deteriorated the fouling condition [12]. The effect of high temperatures, on the other hand, was not consistent. They could increase or decrease membrane scaling, foulant sizes, and cake resistance.

Low feedwater/operating temperature also caused severe fouling conditions and thick fouling layers, leading to high TMP and changes in impurity retention rates for membranes used for water treatment or desalination.

## 3. Temperature Impacts on Membrane Reactor Performance 

For MBRs, temperature is understood to be a vital factor influencing bioreactions so as to impact the performance of the MBRs, but some findings are inconsistent. For example, Chae and Shin [34] reported that when municipal wastewater was treated between 13 °C and 25 °C in a vertical submerged MBR, there was no distinction in pollutant removal efficiency among different temperature environments, with all acceptable removal efficiencies achieved. Another article examined ten pilot and full-scale MBRs treating municipal wastewater, showing that the temperatures of 9.7 °C to 27.4 °C did not affect activated sludge’s apparent viscosity [35].

However, more researchers found that temperature variation did impact membrane performance in multiple respects. Temperatures impacted the membrane permeability by more than changing aqueous viscosity [19]. For instance, the operation at a high temperature of 45 °C not only caused a decrease in biomass quantity and settleability and increased supernatant turbidity compared to 25 °C and 35 °C but also increased SMP production with decreasing EPS production in the reactor. Interestingly, COD removal efficiencies by biological degradation and filtration remarkably declined at the high temperature (45 °C), accompanied by the increased TMP and backwash pressure during the operation. Thus, the overall performance of the MBR deteriorated at 45 °C in treating synthetic-municipal wastewater [36]. On the other hand, Zheng et al. [37] reported that biodegradation was boosted during summertime, and the cold weather in the winter prompted slightly better antibiotic rejection than warm temperatures in MBRs. This might be related to membrane pore size shrinkage in cold temperatures, as discussed in later sections.

Plevri et al. [38] suggested that even temperature variation between 14 °C and 26 °C could dramatically influence the efficiency of a lab-scale AnMBR in treating municipal wastewater. They found that with two days of hydraulic retention time (HRT), the effluent COD concentrations during wintertime were almost twice that in the summertime. Furthermore, a tertiary ultrafiltration system treating the effluent of a sequencing batch reactor (SBR) had lower hydraulically irreversible permeability when the operating temperature dropped from 20 °C to 14 °C and 8 °C. The lower temperatures narrowed the membrane pore sizes, which led to intercepting a significantly high amount of organic matter, while increased water viscosity played the leading role in decreasing hydraulically irreversible permeability [16]. 

In membrane filtration, membrane flux and/or permeability are the decisive parameters for filtration performance. With a bench-scale cross-flow FO system, the temperature increasing from 20 °C to 40 °C promoted membrane water flux increase because of the increased thermal convection and decreased water viscosity [39]. In a fertilizer-driven FO reactor, a reduced viscosity, raised water flux, declined reverse ion flux, and increased specific reverse ion flux were revealed at 45 °C, in which the declined reverse salt flux can mitigate fouling potential [40]. Table 2 presents the collection of articles on the temperature impacts on membrane flux/permeability and filtration performance.

Overall, the literature indicates that temperature influences the performance of membrane filtration operations. As expected, the decrease in membrane flux and/or permeability was observed mainly under the lower operating temperatures that also caused slow biodegradation for the bioreactor. Operating above room temperature could result in lower water viscosity, TMP, and backwash pressure, and vice versa.

## 4. Temperature Impacts on Membrane Structure and Integrity

Only a few studies have been presented on temperature impacts on the structure and integrity of membranes. Table 3 summarizes the published research findings on the temperature effects on membrane integrity and structure in membrane filtration. Membrane materials could encounter physical and chemical changes when water/operating temperature changes. For example, the low temperature of −10 °C compared to 20 °C could reduce PVDF material’s porosity [41]. The membrane with lower porosity has lower membrane flux and higher TMP during the operation, which are vital for evaluating the membrane’s performance.

Pore size change is the microscopic manifestation of the membrane structure distinction under different temperatures. Sharma et al. [19] pioneeringly revealed the pore size differences for nanofiltration membranes in various thermal conditions. They found that the rejection efficiencies of intermediate-size solute molecules, such as dextrose, ethanol, ethylene glycol, glycerol, t-butyl alcohol, and xylose, increased with decreasing temperatures. Furthermore, the average pore sizes of the two commercial nanofiltration membranes were increased by 21% and 12% when the temperature varied from 5 to 41 °C [19]. In another nanofiltration study, a pore-hindrance model showed that the membrane pores of a polyamide membrane were enlarged by 13% when the temperatures were elevated from 20 °C to 40 °C. The authors also indicated that the increased membrane pore size and solute diffusion adversely impacted the pollutant rejection, and the membrane pore sizes were not correlated with the charge repulsion effect under changing temperatures [42]. 

Xu et al. [43] elucidated a similar trend with the membrane pore size variation under different temperature conditions (5, 10, 15, 20, 25 °C), and they found that the two kinds of membranes, loose and tight nanofiltration membranes, showed 14% and 10% pore size shrinkages and 42% and 50% pure water permeability loss, respectively, when the curing temperatures were changed from 25 °C to 5 °C. Lower temperatures increased the rejection of neutrally and positively charged micro-substances. However, the temperature decreases (5–25 °C) had no influence on the rejection of negatively charged micro-pollutants, such as clofibric acid, nalidixic acid, ibuprofen, mefenamic acid, diclofenac, indomethacin, bezafibrate, and candesartan, because the negative electrostatic repulsion compensated for the weaker hindrance induced by pore expansion in higher temperatures between the membrane and contaminants, both with negative charges [43]. In addition, Xiao et al. [44] applied pore-filling of several polymers on the PVDF membrane to create a sensitive temperature-responsive membrane that changed water fluxes up to 15 times between 30 °C and 34 °C. The Scanning Electron Microscope (SEM) images showed distinctive pore sizes at 25 °C and 40 °C.

Cui et al. [6] conducted the first study exploring the membrane structures impacted by an extremely cold temperature at 0.3 °C and revealed that the cold source water temperature could deteriorate the membrane performance, e.g., the shrinkage of the membrane pores. Another comparative research by Tikka et al. [7] also reported the compromise of membrane filtration performance and membrane structure in extremely cold water. A recent study of the influence of cold source water temperature (0.3, 5, and 10 °C) on dynamic changes of membrane structure and the effectiveness of subsequent recovery treatment using warm water revealed that the colder the water temperature, the faster and greater the reduction in membrane pores. Shrunken membrane pores could negatively affect membrane filtration processes. The recovery treatment at 35 °C could fully recover the membrane structure [45]. On the other hand, there are very limited studies on the impact of temperature on the mechanical strength of membranes in drinking water and wastewater treatment. Cui et al. [6] found out that the break strength of hollow fiber PVDF membranes had no statistically significant difference between the virgin membrane operated at room temperature and the membranes operated at a cold temperature (0.3 °C) after about three months.

Membrane integrity under low operating temperatures is rarely researched. Farahbakhsh and Smith [14] were the first to report that low temperature reduced the diffusive air flow rates and the pressure decay rates of the microfiltration membrane, and an extremely low water temperature (around 0 °C) might conceal membrane defection. 

## 5. Impacts of Temperature on Membrane Cleaning

Membrane cleaning is necessary to maintain the filtration flux and restore the performance of fouled membranes during membrane filtration operations. Membrane cleaning consists of physical and chemical cleanings. Chemical cleaning utilizes the chemical properties of cleaning agents, such as oxidation, acidity, alkalinity, and chelation, to decompose, dissolve, and detach the foulants adhering to the fouled membrane surface and inside pores [49].

Cleaning temperatures might not affect some membrane properties. For example, the chemical cleaning temperatures of 20 °C to 34 °C had no profound impact on the membrane zeta potential and surface charge, while cleaning agents and pH did pronouncedly [50]. However, temperature primarily influences the chemical reaction kinetics and therefore affects the rate and effectiveness of chemical cleaning. Increasing the membrane cleaning temperature generally improved the cleaning effectiveness by promoting mass transport processes and the solubility of solids [51]. 

Chen et al. [52] conducted factorial designs to explore the main factors influencing membrane cleaning with ultrafiltration (UF) and reverse osmosis (RO) membranes in municipal wastewater treatment and showed that the cleaning temperature of 50 °C significantly facilitated the chemical cleaning efficiencies as compared to 25 °C. They found that the chemical cleaning at 50 °C mitigated the importance of chemical dosages, and the high cleaning efficiencies could still be achieved at the elevated temperature of 50 °C with the low concentration of the cleaning agent [52]. In another study, Almecija et al. [53] examined a ceramic membrane implemented for protein separation, and the membrane was cleaned at different temperatures of 30, 50, and 60 °C. The optimal temperature for membrane chemical cleaning and recovery was 50 °C while cleaning at 30 °C developed irreversible fouling around the first filtration cycle, and 60 °C cleaning deteriorated and eroded the membrane pores. Rabuni et al. [54] reported that high temperature (50 °C) chemical cleaning incurs a higher flux recovery rate with a bench-scale PVDF ultrafiltration membrane filtration. They revealed that at 50 °C, flux recovery rates could be more than 100% compared to those at 25 °C, potentially resulting from the membrane property alteration and degradation. However, the authors failed to consider that the increased flux could be the effect of pore expansion, which is potentially invertible.

Membrane chemical cleaning not only restores the permeability and performance of membranes but also generates by-products. The cleaning temperature is one of the influential factors for by-product formation [55,56]. For example, NaOCl was applied to clean an ultrafiltration membrane fouled with an algal solution, and the increment of cleaning temperatures from 15 °C to 25 and 35 °C significantly boosted the generation of halogenated by-products, such as dichloroacetic acid (DCAA), trichloronitromethane (TCNM), and trichloromethane (TCM) [55]. On the other hand, Wang et al. [56] suggested that the different temperatures could stimulate or weaken the generation of cleaning by-products. They found that the concentrations of trihalomethanes (THMs) and haloacetic acids (HAAs) at 35 °C were 168% and 248% higher than those at 15 °C, respectively, during the membrane chemical cleaning process. In comparison, haloacetonitriles (HANs) and haloketones (HKs) were 75% and 35% less in the corresponding circumstances, respectively [56].

The cleaning temperature cannot be infinitely increased as the membrane materials have specific tolerances for high temperatures. Thus, the best cleaning temperature is not necessarily higher [57,58]. For example, a polyamide membrane used to treat industrial wastewater was cleaned at 15 °C, 25 °C, 35 °C, and 45 °C in the research conducted by Madaeni and Samieirad [57]. Their experimental results revealed that a low cleaning temperature of 15 °C had a low chemical cleaning efficiency in flux recovery. In contrast, cleaning at 45 °C presented an identical cleaning efficiency as that at 35 °C [57]. Therefore, it suggests that applying membrane cleaning temperatures higher than 35 °C is unnecessary, and the optimized cleaning temperature should be determined to balance the membrane cleaning efficiency and energy consumption in industrial applications. However, the experiment of a cellulose acetate flat-sheet microfiltration membrane fouled with *Chlorella* cells revealed a different optimal cleaning temperature. Ahmad et al. [58] reported that after the third time cleaning the fouled membrane at 60 °C with NaOCl as the cleaning agent, the filtration flux decreased by 7%, while the decrements were 52%, 38%, and 17% with cleaning temperatures of 25 °C, 40 °C, and 80 °C, respectively. Therefore, the authors argued that 60 °C was the best chemical cleaning temperature for the *Chlorella* cell fouled membrane.

Additionally, Simon et al. [59] researched how cleaning water temperature influences the virgin membrane during chemical cleaning. They revealed no distinct temperature impact on the virgin membrane at various cleaning temperatures (20, 35, 50 °C), which was consistent with Al-Amoudi et al.’s [50] result. Simon et al. [59] also found that caustic and acidic cleanings at elevated temperatures significantly increased the membrane surface roughness, which resulted in higher hydrophobicity. The virgin membranes cleaned with citric acid exhibited lower permeability, while caustic-cleaned virgin membranes achieved a higher permeability at 50 °C. Chemical cleaning at excessive temperatures with ethylenediaminetetraacetic-acid (EDTA), sodium dodecyl sulphate (SDS), or citric acid resulted in a better rejection of pharmaceutically active compounds and inorganic salts for membranes, but cleaning with caustic did not.

A limited study has been conducted on the impacts of low membrane cleaning temperatures on membrane filtration plants operating in cold regions during winter. One study by Woo et al. [60] indicated that the chemical cleaning at 2 °C had an 11.6% lower permeation recovery rate than at 23 °C.

Besides chemical cleaning, physical cleaning is more routine in daily operation and maintenance for membrane water treatment plants. Physical cleaning employs hydraulic shear force to physically separate the connections between foulants and the membrane, and the detached foulants fall back into the bulk wash water. Temperature variation also has an impact on the effectiveness of physical cleaning. Zhao and Zou [61] reported that although more severe membrane fouling occurred at the warm temperatures of 35 °C and 45 °C, warm cleaning water at respective temperatures achieved greater efficiency than 25 °C during physical cleaning in a FO desalination system. In an MBR treating synthetic wastewater, Lintzos et al. [62] discovered that increasing backwash temperatures to 38 °C presented better performance and prolonged the intervals of physical cleanings. The article also elucidated that higher backwash water temperatures led to much lower permeability decline rates, though the low-temperature (8 °C) cleaning did not compromise the effluent quality [62]. In addition, Hube et al. [63] also claimed that compared to 25 °C, physical cleaning at 50 °C significantly mitigated intermediate pore blocking and physically irreversible membrane fouling with the filtration of the effluent of the primary settling process from a wastewater treatment plant. Cui et al. [6] and Tikka et al. [7] both reported that membrane structure could be altered during the cleaning at different water temperatures. For example, after treating the cold water at 0.3 °C, the deteriorated membrane structures were recovered after cleaning with warm water (23, 35 °C), and cleaning at 35 °C recovered more membrane permeability.

Table 4 summarizes the impacts of cleaning water temperature on membrane cleaning.

In a general summary of the effect of temperature on membrane separation performance, as compared to the membrane filtration performance under different application situations at room temperature (about 20 °C), a colder water temperature would lead to a decreased membrane flux and/or permeability [6,7,12,20,21,22,28], an increased membrane fouling rate [12,20,28,43], a shrinkage of membrane pore sizes [6,7,45], and reduced membrane chemical cleaning efficiency [52,58,60]. On the other hand, a warmer temperature (in the mesophilic temperature range) would reverse the trends of membrane performance caused by colder temperatures [6,7,39,40,44]. 

## 6. Research Gaps, Challenges, and Opportunities

Although many studies on the impact of temperature on membrane systems have been published, and a good understanding of the role of temperature in determining the efficiency and performance of membrane systems in drinking water and wastewater treatment has been achieved, the literature shows that the detrimental impact of cold temperatures, such as the extremely cold temperature in the winter season in the high latitude regions, has not been well studied. Remarkably, there is a shortage of strategies to abate the effect of extremely cold temperatures on the performance of membrane systems in those cold regions. Previous studies on the cold temperature effect on membrane systems mainly focused on the changes in sludge properties and solution chemistry, such as SMP and NOM, and explained the deteriorated membrane performance by these changes, but the potential of membrane structure under cold water temperature has not received much attention. Thus, further studies on the effect of cold temperature on membrane structure and integrity are highly desirable. Of particular interest are the strategies to recover the reduced membrane pore sizes caused by extremely cold temperatures. Some potential strategies include using warm chemical solutions during routine maintenance and backwash cleaning. These strategies need an in-depth investigation. Another approach would involve designing new membrane materials that would be less sensitive to temperature change to maintain membrane structure while operating in cold temperatures. 

In MBRs, cold temperature affects not only the biokinetics of biodegradation but also the membrane structure and fouling rate. However, the interrelationships among the temperature, sludge properties, biological performance, and membrane performance have not been established. Thus, an in-depth analysis of the existing literature focusing on these aspects, systematic experimental studies of MBRs under these conditions, and complete characterization of influent and sludge properties, membrane fouling and structure, and biological performance, are highly desirable. Additionally, the number of studies of various MBRs, including aerobic MBRs and anaerobic MBRs, for wastewater treatment under psychrophilic temperatures is limited. Further studies in this area are needed, considering that several industrial wastewaters, such as malting and brewery wastewater, are discharged under psychrophilic temperatures and are suitable for MBR treatment.

Mathematical modelling plays a vital role in designing and optimizing membrane systems for drinking water and wastewater treatment. Although some proposed mathematical models describe the impact of processes and environmental conditions on the performance of membrane systems, there are only limited studies modelling the impact of temperature (cold to hot temperatures) on the dynamic changes in membrane structure (pore sizes) and chemical cleaning efficiency. However, in recent years, some researchers have mathematically studied the effect of temperature on membrane structure by using different mechanisms to establish the mathematical models, such as models based on heat transfer and energy balance [48], different forces [46], and the theory of thermoelasticity [47]. These studies on the mathematical modelling of membrane performance and structure provide new insight into how process variables affect membrane structure and eventually membrane performance. These models can be used to optimize the operation and performance of membrane filtration plants and thus deserve more in-depth studies in this area. Thus, mathematical models incorporating the membrane materials’ properties, mechanics, heat transfer, and temperature are highly sought to design and optimize the performance of membrane systems and chemical cleaning.

## 7. Conclusions

Seasonal temperature variations could be encountered in many membrane filtration plants for water and wastewater treatments, depending on the geographical locations of these plants. Feedwater/operating temperature changes could significantly impact the membrane structure, performance, fouling, and cleaning of membrane filtration systems. In most cases, cold temperatures arouse more unfavourable conditions in both biological reactions and membrane performances during membrane filtration operations. Strategies to minimize the impact of cold temperature and mathematical modelling of dynamic membrane structure changes are called for further studies. Although research scientists and engineers are still working on compensating and offsetting the adverse effects of cold feedwater, the current findings are insufficient to direct industrial operations to cope with the problem. Nevertheless, limited research has been completed, and temperature effects should be neutralized or even manipulated to benefit membrane technology in drinking water and wastewater treatments.

## Figures and Tables

**Table 1:** Summary of the impacts of temperature on membrane fouling and foulant properties.

Membrane Type and Material	Reactor	Temperature Examined (°C)	Summary	Reference
Hollow fibre polyvinylidene fluoride (PVDF) membrane	Full-scale municipal MBR	8–24	Temperature decreases caused decreased sludge stabilization, settle ability, and dewaterability adjustments in cleaning frequencies (or fouling rate)	[15]
RO membrane	Bench-scale reactor	15, 25, 35	Temperature decreases caused increased TMP increased solute rejection	[21,22]
Flat sheet polyethersulfone (PES) membrane	Pilot-scale MBR	8–26	Temperature decreases caused increased EPS, COD, polysaccharides, and protein in supernatants decreased sludge settle ability, dewaterability increased membrane fouling rates	[23]
PE microfiltration (MF) membrane	Bench-scale PAC-MBRs	10, 20		[24]
Hollow fibre PVDF membrane	Pilot-scale MBR	8.7–19.7		[25]
Hollow fibre PVDF MF membrane	Pilot-scale SAnMBR with real municipal wastewater as influent	15, 20, 25		[20]
Flat-sheet PVDF membrane	Lab-scale SAnMBR	37, 42, 47, 45, 50, 55	High-temperature shock (37 to 42 °C, 37 to 47 °C, 45 to 50 °C, 45 to 55 °C) caused biogas production smaller pieces of sludge flocs temporarily decreased and then increased fouling resistance no influence on microbial community structure diverse microbial species and richness	[17,18]
Flat-sheet PVDF membrane	Pilot-scale MBR	7, 15, 25	Mechanisms of membrane resistance increase when the temperature decreased: water viscosity increase sludge deflocculation decreased back transport velocity restrained COD biodegradation	[12]
FO membrane	Lab-scale reactor	20, 35, 50	Temperature increases caused increased flux rates less fouling	[26]
Spiral-wound RO membrane	Lab-scale reactor	10, 20, 30	Temperature decreases caused decreased biomass growth rates decreased salt retention ability thickened fouling layer decreased permeation fluxes	[27]
Hollow fibre PVDF ultrafiltration (UF) membrane	Bench-scale reactor	13–20, 20–30		[28]
Tubular ceramic UF membrane	Lab-scale reactor	5, 20, 35	Temperature decreases caused increased flux decreasing rates increased NOM retention rates reduced hydraulic and chemical cleaning efficiencies	[11]
Flat sheet polytetrafluoroethylene (PTFE) membrane	Bench-scale direct contact membrane distillation	40, 60	High temperature (60 °C) caused significant flux decline microbial fouling structure changes	[29]
Ceramic UF membrane	Bench-scale reactor	5, 20, 35	Temperature decreases induced more irreversible foulants Both higher viscosity and exacerbated fouling conditions contributed half to the increase in TMP when temperature decreased	[13]
GO-coated calcium sulphate RO membrane	Bench-scale RO desalination	5, 15, 25, 35	Temperature increases caused severe membrane scaling and high resistance different scaling morphologies	[30]
PTFE microfiltration membrane	Bench-scale direct contact membrane distillation	50, 60, 70, 80	When the temperature increased, calcium sulphate was more likely to form a sizeable needle-shaped crystal and severely foul the membrane	[31]
Hollow fibre PVDF MF membrane	Lava stone biocarrier facilitated gravity-driven membrane reactors	8, 22	Temperature increases caused foulants to accumulate more and have a larger size no change in cake resistance without cleaning increased cake resistance with periodic cleaning no influence on foulant content and permeate quality	[32]
Zeeweed-1000 hollow fibre UF membrane	Bench-scale sequencing batch reactors (SBRs) with municipal wastewater as influent	8, 14, 20	Decreasing temperatures increase membrane resistance from fouling and intrinsic resistance.	[33]

**Table 2:** Summary of the impacts of temperature on filtration and membrane performances.

Membrane Type and Material	Reactor	Temperature (°C)	Summary	Reference
PES hollow fibre MF membrane	Lab-scale submerged MBR treating synthetic-municipal wastewater	25, 35, 45	Temperature increases caused decreased biomass quantity and sludge settleability increased supernatant turbidity increased SMP, decreased EPS decreased COD removal rates decreased TMP and backwash pressures decreased overall performance	[36]
Asymmetric cellulose triacetate FO membrane and thin-film composite polyamide FO membrane	Bench-scale cross-flow FO system	20, 40	Increased temperatures increased membrane flux	[39]
Unknown	Full-scale wastewater treatment plant MBR	spring, summer, autumn, winter	Low temperatures during wintertime caused slow biodegradation slightly better antibiotic rejection	[37]
FO membrane	Lab-scale fertilizer-driven FO	25, 30, 35	Temperature increases caused decreased water viscosity decreased reverse salt fluxes increased water fluxes increased specific reverse salt fluxes	[40]
Flat sheet membrane	Lab-scale submerged AnMBR with real wastewater	18 ± 4 (winter), 24 ± 3 (summer)	Temperature decreases increased effluent CODs with different HRTs	[38]
Zeeweed-1000 hollow-fibre ultrafiltration membrane	Filtration of the effluent of bench-scale SBRs treating real municipal wastewater	8, 14, 20	Temperature drop caused the decreasing hydraulically irreversible permeability by altering influent organic matter features increasing water viscosity narrowing membrane pores membrane foulants’ interactions	[16]

**Table 3:** Summary of the impacts of temperature on structure and integrity.

Membrane Type and Material	Reactor	Temperature (°C)	Summary	Reference
Zeeweed-500 hollow fibre MF membrane	Lab-scale filtration	0–30	Temperature decreases reduced the diffusive air flow rates reduced the pressure decay rates caused membrane defection	[14]
Two commercial polyamide thin-film nanofiltration (NF) membranes	Lab-scale filtration	5, 15, 23, 35, 41	Temperature decreases caused increased reflection rate of intermediate-size solute molecules membrane pore shrinkage no impact on charge repulsion permeability loss increased rejection of neutrally and positively charged micro-substances	[19]
NF270 flat-sheet NF membranes	Lab-scale filtration	20, 30, 40		[42]
A loose and a tight NF membrane	Lab-scale cross-flow filtration	5, 10, 15, 20, 25		[43]
PVDF MF membranes and flatsheet PNIPAAm–PVDF membrane	Lab-scale filtration	25, 30, 34, 40, 48	Pore-filling several polymers on the PVDF membrane formed a sensitive temperature-responsive membrane. The membrane had dramatically distinctive pore sizes between 25 °C and 40 °C The permeation fluxes varied up to 15 times between 30 °C and 34 °C	[44]
PVDF hollow fibre MF membrane	Lab-scale filtration	0.3, 21	Extremely cold water temperatures at 0.3 °C deteriorated membrane performance and led to membrane pore shrinkage. The deterioration could be almost recovered at room temperature A higher remediated temperature recovered more permeability for the membrane	[6]
PVDF hollow fibre MF membrane	Lab-scale filtration	0.3, 21, 35		[7]
PVDF hollow fibre MF membrane	Lab-scale filtration	0.3, 5, 10, 21, 35	The colder the water temperature, the faster and greater the reduction in membrane pores. The recovery treatment at 35 °C could fully recover membrane structure.	[45]
Lot M38 & Lot M39 Membrane	Lab-scale experimental and modelling studies	room temperature, 60	Membrane pore size increased with time with thermal treatment. A non-linear mathematical model based on the consideration of different forces was proposed to describe the change in membrane pore size with thermal treatment.	[46]
Hybrid membrane of poly (N-isopropylacrylamide), (PNIPAM) within PTFE	Lab-scale experimental and modelling studies	20, 40 (temperature cycle)	Membrane pore opening and closing dynamics followed the temperature cycle. Mathematical models based on the theory of thermoelasticity were developed to model the thermo-response of membrane pore size change in temperature cycles.	[47]
PTFE membrane	Lab-scale experimental and modelling studies	17, 60, 70, 80	As the temperature increased, the pore size gradually increased. Mathematical models based on heat transfer and energy balance were developed to describe the change in membrane pore sizes with temperature change and thermal treatment.	[48]

**Table 4:** Summary of the impacts of temperature on cleaning.

Membrane Type and Material	Fouled Membrane Origin	Cleaning Method	Temperature (°C)	Summary	Reference
Spiral- wound thin film PES UF and Polyamide RO membranes from Fluid Systems	Filtrated by secondary effluent collected from a local sewage treatment plant	Physical: forward flush, backwash Chemical: TriClean 212F	25, 50	Increased cleaning temperatures caused: increased cleaning efficiencies chemical dosage economy at 50 °C, flux recovery rates can be more than 100%	[52]
Flat sheet cellulose triacetate (CTA) FO membranes	Treating actual and simulated brackish water	Physical	25, 35, 45		[61]
Flat sheet UF PVDF membrane	Fouled with bovine serum albumin (BSA)	Chemical: NaOH, NaOCl	25, 50		[54]
Three commercial NF membranes	Saline water desalination	Chemical: HCl; NaOH; SDS; Mixed agent of EDTA, trisodium phosphate (TSP) and sodium tripolyphosphate (STP); NaOH followed by HCl	20, 23, 25, 30, 34	Cleaning temperature had no impact on membrane surface charge and zeta potential.	[50]
Tubular ceramic Céram Inside membrane made of ZrO_2_–TiO_2_	Fouled by protein solution containing β-lactoglobulin and bovine serum albumin	Chemical: sodium hydroxide and sodium dodecyl sulphate solution	30, 50, 60	50 °C was the optimal temperature 30 °C developed irreversible fouling 60 °C eroded the membrane	[53]
FT-30 polyamide membrane	Treated industrial wastewater	Chemical: HCl, HNO_3_, H_2_SO_4_, NaOH, EDTA, SDS	15, 25, 35, 45	35 and 45 °C had the same cleaning efficiency Lower cleaning temperatures recovered fewer fluxes	[57]
PVDF hollow fibre membrane	Pilot plant treating river water	Chemical: NaOH, NaOCl, H_2_SO_4_, HNO_3_, citric acid, and oxalic acid	2, 23		[60]
NF270 NF membrane with “a semi-aromatic piperazine-based polyamide skin layer on top of a microporous” polysulphone (PS) backing layer	Virgin membrane	Chemical: citric acid, NaOH, EDTA, and SDS	20, 35, 50	Temperatures had no impact on the virgin membrane cleaning Raised cleaning temperatures increased membrane roughness and hydrophobicity Different cleaning agents combined with various temperatures expressed distinctive effects	[59]
Cellulose acetate flat-sheet MF membrane	Fouled with Chlorella cells	Chemical: NaOH, NaOCl, nitric acid, and citric acid	25, 40, 60, 80	60 °C was the optimal temperature Other temperatures had lower flux recovery rates	[58]
Hollow fibre UF R-PVDF membrane	Fouled with synthetic wastewater	Physical: backwash	8, 18, 28, 38	Increased backwash water temperatures presented better performance prolonged the intervals of physical cleanings led to much lower permeability-decreasing rates	[62]
Flat-sheet PES ultrafiltration membrane	Fouled with algal solution	Chemical: NaOCl	15, 25, 35	Increased cleaning temperatures significantly boosted the generation of halogenated by-products.	[55]
PVDF ultrafiltration membrane	Fouled with real wastewater	Physical	25, 50	High cleaning temperature mitigated intermediate pore blocking and physically irreversible fouling.	[63]
PVP/SiO_2_ modified hollow fibre PVDF ultrafiltration membrane	Fouled with simulated domestic sewage	Chemical: NaOCl	15, 25, 35	Cleaning temperatures could impact the generation of disinfecting by-products.	[56]

## Data Availability

Data sharing not applicable. No new data were created or analyzed in this study.

## Abbreviations

AnMBR	Anaerobic Membrane Bioreactor
BSA	Bovine Serum Albumin
COD(s)	Chemical Oxygen Demand(s)
CST	Capillary Suction Time
CTA	Cellulose Triacetate
DCAA	Dichloroacetic Acid
DSVI	Diluted Sludge Volume Index
EDTA	Ethylenediaminetetraacetic Acid
EPS	Extracellular Polymeric Substances
FO	Forward Osmosis
GO	Graphene Oxide
HAAs	Haloacetic Acids
HANs	Haloacetonitriles
HKs	Haloketones
HRT(s)	Hydraulic Retention Time(s)
MBR(s)	Membrane Bioreactor(s)
MF	Microfiltration
NF	Nanofiltration
NOM	Natural Organic Matter
PAC	Poly Aluminum Chloride
PES	Polyethersulfone
PNIPAAm	Poly(N-Isopropylacrylamide)
PS	Polysulphone
PTFE	Polytetrafluoroethylene
PVDF	Polyvinylidene Fluoride
PVP	Polyvinyl Pyrrolidone
RO	Reverse Osmosis
SAnMBR	Submerged Anaerobic Membrane Bioreactor
SBR(s)	Sequencing Batch Reactor(s)
SDS	Sodium Dodecyl Sulphate
SEM	Scanning Electron Microscope
SMP	Soluble Microbial Products
STP	Sodium Tripolyphosphate
TCM	Trichloromethane
TCNM	Trichloronitromethane
THMs	Trihalomethanes
TMP	Transmembrane Pressure
TSP	Trisodium Phosphate
UF	Ultrafiltration
